# Misinterpretations of P-values and statistical tests persists among researchers and professionals working with statistics and epidemiology

**DOI:** 10.48101/ujms.v127.8760

**Published:** 2022-08-04

**Authors:** Per Lytsy, Mikael Hartman, Ronnie Pingel

**Affiliations:** a Department of Public Health and Caring Sciences, University of Uppsala, Uppsala, Sweden; b Independent Researcher; c Department of Statistics, University of Uppsala, Uppsala, Sweden

**Keywords:** Statistical inference, null hypothesis significance testing, statistics, frequentist, P-value

## Abstract

**Background:**

The aim was to investigate inferences of statistically significant test results among persons with more or less statistical education and research experience.

**Methods:**

A total of 75 doctoral students and 64 statisticians/epidemiologist responded to a web questionnaire about inferences of statistically significant findings. Participants were asked about their education and research experience, and also whether a ‘statistically significant’ test result (*P* = 0.024, α-level 0.05) could be inferred as proof or probability statements about the truth or falsehood of the null hypothesis (H_0_) and the alternative hypothesis (H_1_).

**Results:**

Almost all participants reported having a university degree, and among statisticians/epidemiologist, most reported having a university degree in statistics and were working professionally with statistics. Overall, 9.4% of statisticians/epidemiologist and 24.0% of doctoral students responded that the statistically significant finding proved that H_0_ is not true, and 73.4% of statisticians/epidemiologists and 53.3% of doctoral students responded that the statistically significant finding indicated that H_0_ is improbable. Corresponding numbers about inferences about the alternative hypothesis (H_1_) were 12.0% and 6.2% about proving H_1_ being true and 62.7 and 62.5% for the conclusion that H_1_ is probable. Correct inferences to both questions, which is that a statistically significant finding cannot be inferred as either proof or a measure of a hypothesis’ probability, were given by 10.7% of doctoral students and 12.5% of statisticians/epidemiologists.

**Conclusions:**

Misinterpretation of *P*-values and statistically significant test results persists also among persons who have substantial statistical education and who work professionally with statistics.

## Introduction

Researchers typically test their research question by formulating a hypothesis and then quantitatively test it by performing a Null Hypothesis Statistical Test (NHST) (1). While there are many different types of statically tests within the NHST framework, they all share a common ground: by assuming a specific hypothesis (typically denoted the null) to be true, it is possible to investigate whether the observed data are compatible or not align with the given model. This article investigates what inferential conclusions researchers in training and persons familiar with statistics find justifiable facing a statistically significant test finding.

Inferential statistics is inherently associated with uncertainty, which may be further categorised as being different kinds. It is possible that the data at hand, i.e. the sample that is analysed, are unusual, and a poor representation of the phenomenon investigated simply because of systematic errors and biases, caused, for example, by poor study design and scientific misconduct. It is also possible that the sample is unrepresentative of the phenomenon of interest because of sampling errors (random errors). The risk of sampling errors may be reduced, but never eliminated, by the use of sufficiently large samples. Other uncertainties have to do with the tested statistical model, usually easy to describe in words or mathematical terms, however, based on a priori assumptions, which sometimes are unrealistic, unjustified and practically difficult to verify (2, 3).

This study is about yet another kind of uncertainty, which has to do with inferences of the results from the NHST. This uncertainty is not a characteristic of the tested model or the data, rather it is about logic and the cognitive abilities of the interpreter, as it has to do with knowledge about proper interpretation of statistical test result. The debate over misapplication and misinterpretation of test results is old (4, 5), and in the past years, it has been fuelled with the debate of a replication crisis and promotion of good statistical conduct (6). In order to grasp the controversy, one needs to understand that the NHST is a procedure including two different tests, the significance test and the hypothesis test, which have different aims and are based on different theories. Critics claim that these tests are incoherent, and that the hybrid procedure used has several methodological flaws (4, 5, 7–11). (For a longer list of references on the topic, please see the article by Greenland et al. (2).) Defenders of the NHST have argued that the problem is not about the method itself, rather its misuse or abuse, potentially manageable by education and better practice.

There have been many attempts to correct misunderstandings and misuse of statistical inference. One such is a statement about statistical significance and *P*-values, published in 2016 by the American Statistical Association (ASA) (12). The statement aimed at, in a non-technical way, highlighting six principles about inference, all having widespread consensus in the statistical community, that could improve the conduct or interpretation of quantitative science (12, 13). Reviewing and explaining all the inferential mistakes that commonly occur when interpreting the results from null hypothesis significance testing is beyond the scope of this article, but some points raised by the ASA-statement are central to the present study.

First (point 2 in the ASA-statement): a *P*-value does not measure the probability that the studied hypothesis (which is the null hypothesis, hereafter denoted H_0_) is true. It cannot do that, since the test is based on the *premises that H*_*0*_ *is true*. Thus, it is not possible to infer, from a *P*-value, the truthfulness or the probability of a hypothesis, whether that is the tested hypothesis (H_0_) or any other hypothesis, such as the alternative (hereafter denoted H_1_). The *P*-value, in the way it is defined and calculated, is simply not a measure in favour of any hypothesis.

Second (point 3 in ASA-statement): scientific conclusions should not be based only on whether a *P*-value passes a specific threshold using mechanical rules such as *P* < 0.05. The authors write that ‘the wide spread use of “statistical significance” (generally interpreted as ‘*P* ≤ 0.05’) as a license for making a claim of a scientific finding (or implied truth) leads to considerable distortion of the scientific process’. The misuse and misunderstandings have led to suggestions to abandon the practice of classifying findings as ‘statistically significant’ (14, 15).

Third (point 6 in ASA-statement): by itself, a *P*-value does not provide a good measure of evidence regarding a model or hypothesis. This is because the *P*-value is not only a measure based on the observations but also more extreme data representing observations that never occurred, under the given model/hypothesis. Also, the likeliness of the null being true may be more or less likely to start with, which is not considered when performing NHST:s (16). For anyone interested in more formal arguments behind these principles, please see the references in the ASA-statement as well as the references cited earlier in the manuscript by Greenland et al. (12).

Over the years, there have been plenty of published papers highlighting these issues and warning against common misinterpretations about *P*-values and results labelled as statistically significant findings (2, 8, 11, 17, 18). There are also studies that empirically have investigated the magnitude of erroneous inferences from statistical test results (5, 9, 19–21). One of the first investing this was Michel Oakes, who, in 1986, reported that almost all of 70 academic psychologists misinterpreted the *P*-value as the probability of the null hypothesis being true. Furthermore, most of them believed that a *P*-value of 0.01 implied a 99% chance of statistically significant future replications (5). Both these interpretations are erroneous.

In 2004, Gigerenzer performed a study with similar results, where a sample of psychology professors, teachers of statistics and students were given a result of a straightforward *t*-test of two independent means and were then asked to state whether none, one or several of six statements of common interpretations of the *P*-value were true or false. The results showed that none of the 44 students, 4 of the 39 professors and lectures not teaching statistics and 6 of the 30 professors and lectures who teach statistics – got all questions right (9).

This present study builds on these previous empirical studies of misunderstandings of statistical inference and explores whether the misunderstandings persist. This study further aims to explore whether having more and less statistical education and scientific experience is associated with correct statistical inferences. A secondary aim arose from a randomised setting of the survey, making it possible to investigate if inferences of results would be affected by earlier questions on inferences of more or less likely findings.

## Material and methods

### Study population, study design and randomisation

An invitation to participate in a study ‘about statistical inference’ was sent out by email to all members of the Swedish Society for Medical Statistics (approximately 200 members) and all members in the Swedish Society for Epidemiology (approximately 100 members) in May 2016. Both these organisations are open for paid membership for anyone interested in medical statistics and epidemiology.

An invitation was also sent to every PhD student (approximately 600 PhD students) at the medical and pharmaceutical faculty at Uppsala University. The invitation procedures were carried out to create two groups: a group of participants with high likeliness of interest and knowledge in the statistical and/or epidemiological field (this group is hereafter denoted ‘statisticians’) and a group of doctoral students assumed to have not only some statistical knowledge/education but also a high likeliness of having at least basic medical training (here after denoted ‘doctoral students’). In total, 75 statisticians/epidemiologists and 94 doctoral students agreed to participate and fulfilled a web-questionnaire, which contained 14 questions. At the start, there was a possibility to choose to answer the questionnaire in either Swedish or English.

There were two main predefined purposes: one included an experimental setting to investigate if responder’s preconception about the plausibility of the tested hypothesis affected their inferences. The other purpose, which is reported in the present article, was to investigate respondent’s views about the legitimate conclusion that can be drawn from a ‘statistically significant’ finding.

The participants were, without their knowledge, allocated to one of two groups using a single-blind procedure in the strata doctoral students and statisticians. Participants in the allocated groups received two slightly different text versions asking them to interpret a statistically significant test result of a randomised placebo-controlled study investigating the effect of a ‘new homeopathic treatment’, whereas the other groups received the exact same text, but phrased as the study was investigating the effect of a ‘new treatment’. The purpose of the different questions was to investigate whether they would be associated with any difference in assessments, on a 0–100 visual analogue scale, of the likeliness that treatment had a true effect. The results of this question will be reported in a separate publication. The experimental setting with groups receiving somewhat different information in one of the questions is of importance in the present study, since it may affect other outcomes. A screen shot of the question about the clinical trial (new homeopathic drug version) is available in Appendix 1.

Since the prior belief in homeopathy as an effective treatment may affect the assessment of the trial results, we asked respondents to a state their view on homeopathy. Respondents received the following information: ‘The claim of homeopathy is that an ultra diluted remedy, to the point where no active ingredient remains, may still have therapeutic properties. The mechanism of action is insofar unclear. Do you find it likely that homeopathy may work?’ Response options were ‘Very unlikely’, ‘Unlikely’, As likely as ‘unlikely’, ‘Likely’ and ‘Very likely’.

### Outcomes

The present study reports the results of two outcome questions that were presented to all participants in the same way after they had responded to question about the clinical trial. The questions were about the legitimate inferences one may conclude about the research hypothesis of interest to the researcher (H_1_) and to the modelled null hypothesis (H_0_), when having a ‘statistically significant test result’.

The following information was given under the headline: *The meaning of statistical significance*: ‘A researcher wanted to test a hypothesis (H_1_) and set up a statistical test with a null hypothesis (H_0_). The significance test resulted in a *P*-value of 0.024 which was lower than the present level of significance (*P* < 0.05), thus, the result was statistically significant’.

The text was followed by two questions: ‘What does the test say about H_0_?’, with the fixed responses: ‘it proves that H_0_ is not true’, ‘it shows that H_0_ is improbable’ and ‘none of the answers above are correct’. The other question, visible on the same web-page, was phrased, ‘What does the test say about H_1_?’ with the fixed responses: ‘it proves that H_1_ is true’, ‘it shows that H_1_ is probable’ and ‘none of the answers above are correct’.

The correct answers, for both questions, are that ‘none of the above answers are correct’. A dichotomised variable of answering both questions correctly vs. not was created.

**Figure 1:**
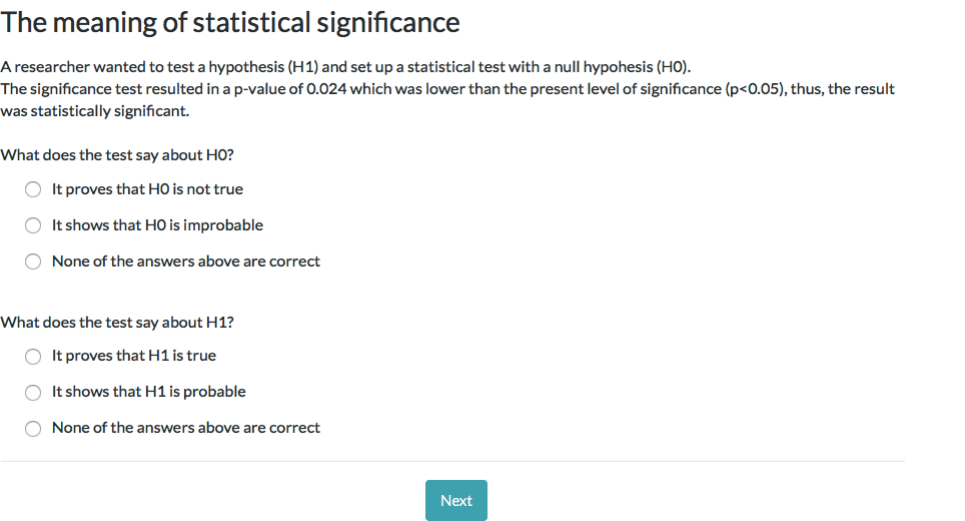
Screenshot of the outcome question.

A screen shot of the text, questions and response-alternatives answers is shown in Figure 1.

### Other data collected

After the outcome questions, the respondents were asked to answer some questions about themselves. Participants were asked about their age, sex and highest level of education (response alternatives: elementary school, senior high school, unfinished university degree, university degree, university degree being a current doctoral student or university degree + an academic degree, PhD or licentiate). Respondents were also asked how much medicine or pharmacology they had studied (response alternatives: none, less than a year, 1 year or more) as well as how much statistics they had studied (response alternatives: none or almost none, 1–4 weeks, 5–20 weeks or 20 weeks or more). There were also questions about whether having a university degree in statistics (yes/no) and whether having a doctoral degree in statistics (yes/no). The respondents were additionally asked if they currently worked professionally with statistics (yes/no) and how may peer-reviewed scientific papers they had authored/co-authored (none, 1–4, 5–20 or more than 20). Respondents were also asked, after they had answered the question about interpreting a treatment/homeopathic treatment, whether they find it likely that homeopathy may work. Fixed responses were very unlikely, unlikely, as likely as unlikely, likely and very likely.

Every question had to be answered to proceed to the next one. It was not possible to go back and change previous answers or to answer the questionnaire twice. At the end, there was a possibility to leave comments about the study and the questions.

No ethical approval was needed as this study did not collect any personal information considered sensitive according to the Swedish law. Participation was voluntary, and responding to the questionnaire was considered giving informed consent as the purpose of publishing the results was clearly stated.

### Data analysis

Descriptive statistics were used to calculate proportions of respondent’s answers to the outcome questions, in the total population and in different strata being: woman or man, statistician/doctoral students, having more than 20 weeks statistical education vs. not, working professionally with statistics or not, have less than five peer-reviewed publications or not, having a PhD-degree or not, and being allocated to different treatment phrasings in the previous question about the clinical trial. Chi square tests were used to test the differences in proportions in strata. Analyses were performed using Stata, version 14.2 (StataCorp, Texas, US). The pre-set alfa-level was set to 5%.

## Results

### Study population characteristics

Of the 169 persons who agreed to participate, 139 (75 doctoral students and 64 statisticians) responded to the two outcome questions, making the overall response rate of randomised participants as 82.2%. There were more women in the doctoral student group, whereas there were more men in the group of statisticians (male respondents: doctoral group 33.3%; statistician group: 62.5%). Almost all participants in both groups reported having a completed university degree. Among statisticians, 78.1% reported having a university degree in statistics and 26.6% also reported having PhD in statistics. Almost one-third of doctoral students responded that they worked professionally with statistic; the corresponding number in the group of statisticians was 90.6%. More doctoral students (78.7%) than statisticians (34.4%) reported having some kind of formal medical education.

The majority of the statistician’s hade authored or co-authored more than five scientific publications in peer-review literature. In summary, the statistician’s group reported, on average, having higher statistical education and more scientific experience, whereas doctoral students reported having somewhat more medical or pharmaceutical education. A summary of the characteristics of the study groups is presented in Table 1.

**Table 1:** Characteristics of study population, grouped as PhD students, statisticians and total.

	PhD students	Statisticians	Total
*n*	75	64	139
Sex, male, % (*n*)	33.3 (25)	62.5 (40)	46.8 (65)
Age, mean (SD)	35.1 (9.0)	48.5 (12.4)	41.3 (12.6)
University degree, % (*i*)	100 (75)	98.4 (63)	99.3 (138)
University degree in statistics, % (*n*)	4.0 (3)	78.1 (50)	38.1 (53)
PhD degree, % (*n*)	9.3 (7)	62.5 (40)	33.8 (47)
PhD degree in statistics, % (*n*)	0 (0)	26.6 (17)	12.2 (17)
Works professionally with statistics	29.3 (22)	90.6 (58)	57.6 (80)
Any formal medical education, % (*n*)	78.7 (59)	34.4 (22)	58.3 (81)
20 weeks or more of statistical education, *n* (%)	16.0 (12)	90.6 (58)	50.4 (70)
Five or more published articles, *n* (%)	17.3 (13)	78.1 (50)	45.3 (63)

### Interpretation of a statistically significant result

The main purpose was to investigate the respondents’ view on what conclusions that are warranted, given a statistically significant test result. Overall, 17.3% believed that a ‘statistically significant’ result *proves* that H_0_ is true, 62.6% that it shows that H_0_ is *improbable* and 20.1% that neither of these alternatives is correct. When asked what the results say about H_1_, 9.4% responded that it *proves* that H_1_ is true, 62.6% that it shows that H_1_ is *probable* and 28.1% that none of these alternatives is correct. Overall, 11.5% gave correct answers to both questions: that is, a significant test result does not warrant any legitimate conclusions about whether either the tested hypothesis (H_0_) or the alternative hypothesis (H_1_) is false, true or probable.

The results in different strata were very much alike the pattern in the total group. There were differences between the doctoral student group and statisticians’ group in terms of inference on H_0_. A lower proportion of statisticians, as compared to doctoral students (9.4% vs. 24.0%), answered that a statistically significant finding proves that H_0_ is not true, and a higher proportion of statisticians (73.4% vs 53.3%) answered that a statistically significant finding indicates that H_0_ is improbable. A similar pattern was seen for persons having a PhD. Persons who were allocated to the group having previously answered a question about the interpretation of a study investigating ‘a new homeopathic treatment’ were less likely to report that a statistically ‘significant finding’ proves that H_1_ is true (4.3 vs 14.5%), and were more likely (17.1% vs 5.8) to answer correctly in both questions about inferences of H_0_ and H_1_. The results, in total and from the stratified analyses, are shown in Table 2.

**Table 2:** Summary of answers to the statement.

Variable	What does the test say about H_0_?				What does the test say about H_1_?			Correct answer to both H_0_ and H_1_
	*n*	It proves that H_0_ is not true	It shows that H_0_ is improbable	None of the answers above are correct	It proves that H_1_ is true	It shows that H_1_ is probable	None of the answers above are correct	
All	139	17.3 (24)	62.6 (87)	20.1 (28)	9.4 (13)	62.6 (87)	28.1 (39)	11.5 (16)
PhD students	75	24.0 (18)	53.3 (40.0)	22.7 (17)	12.0 (9)	62.7 (47)	25.3 (19)	10.7 (8)
Statisticians	64	9.4 (6)	73.4 (47)	17.2 (11)	6.2 (4)	62.5 (40)	31.2 (20)	12.5 (8)
*P*-value		0.030			0.438			0.943
Less than 20 weeks of statistical education	69	23.2 (16)	55.1 (38)	21.7 (15)	11.6 (8)	65.2 (45)	23.2 (16)	10.1 (7)
At least 20 weeks of statistical education	70	11.4 (8)	70.0 (49)	18.6 (13)	7.1 (5)	60.0 (42)	32.9 (23)	12.9 (9)
		0.123			0.360			0.814
Do not work professionally with statistics	59	23.7 (14)	54.2 (32)	22.0 (13)	13.6 (8)	62.7 (37)	23.7 (14)	6.8 (4)
Work professionally with statistics	80	12.5 (10)	68.8 (55)	18.8 (15)	6.2 (5)	62.5 (50)	31.2 (25)	15.0 (12)
		0.149			0.269			0.218
≤ 5 publications	76	19.7 (15)	56.6 (43)	23.7 (18)	11.8 (9)	61.8 (47)	26.3 (20)	11.8 (9)
> 5 publications	63	14.3 (9)	69.8 (44)	15.9 (10)	6.3 (4)	63.5 (40)	30.2 (19)	11.1 (7)
*P*-value		0.272			0.520			1.00
No PhD degree	92	23.9 (22)	56.5 (52)	19.6 (18)	12.0 (11)	64.1 (59)	23.9 (22)	9.8 (9)
PhD degree	47	4.3 (2)	74.5 (35)	21.3 (10)	4.3 (2)	59.6 (28)	36.2 (17)	14.9 (7)
*P*-value		0.014			0.154			0.540
Women	74	20.3 (15)	59.5 (44)	20.3 (15)	10.8 (8)	64.9 (48)	24.3 (18)	12.2 (9)
Men	65	13.8 (9)	66.2 (43)	20.0 (13)	7.7 (5)	60.0 (39)	32.3 (21)	10.8 (7)
*P*-value		0.584			0.528			0.797
Allocation ‘homeopathic treatment’	70	14.3 (10)	60.0 (42)	25.7 (18)	4.3 (3)	60.0 (42)	35.7 (25)	17.1 (12)
Allocation ‘treatment’	69	20.3 (14)	65.2 (45)	14.5 (10)	14.5 (10)	65.3 (45)	20.3 (14)	5.8 (4)
*P*-value		0.218			0.031			0.036

A researcher wanted to test a hypothesis (H_1_) and set up a statistical test with a null hypothesis (H_0_). The significance test resulted in a *P*-value of 0.024, which was lower than the present level of significance (*P* < 0.05); thus, the result was statistically significant. The results are summarised with frequencies and row percent (%) for all respondents and within different strata.

Twenty-one participants took the opportunity to give free text comments on the study. Some were neutral, such as ‘interesting study’ and ‘good luck’. There were some specific comments on the question about inferences of the drug/homeopathic drug, mainly about the need of more information. One comment had direct implications of on the present study, it was: ‘The question about H_1_ is tricky. When we do a test, only H_0_ is tested, and rejected or not rejected…’

## Discussion

This study aimed at investigating if statistical inference misunderstandings persist. The answer is clearly yes, since the majority of responders committed the inferential mistakes often warned about (2, 17) and seen in studies previously (5, 19, 21). The most common inferential mistake was what is often called the *inverse probability fallacy*, which is the false belief that a low *P*-value or a statistically significant result conveys information of the probability of the null hypotheses being true. This fallacy has been known for decades (22), and despite warnings and education, the fallacy seems to persist.

Overall, a little less than one-fifth (17.3%) and a little less than one-tenth (9.4%) responded that the test results ‘proved’ that H_0_ is false or that H_1_ is correct, respectively. Although rather uncommon, these are extraordinary errors. According to the Popperian view of science, no amount of scientific evidence can conclusively *prove* a general proposition, and statistical inference is a practise of deducing conclusions from samples to general populations, which means that statistical evidence can never ‘prove’ anything, only gather evidence. The results (Table 2) show that persons who were more statistically educated and having more research experience were less prone to regard statistically significant findings as proof. However, being more ‘correct’ in that sense seems to come at the cost of other mistakes as a higher proportion of the same group responded that statistically significant findings indicate a low probability of H_0_ being true, whereas this was less clear for conclusions about H_1_.

The experimental allocation, where participants were divided into two groups and received different background information in a question about a clinical trial, was associated with the outcomes. The idea behind the group allocation was to create two situations where respondents were more or less prejudiced towards the likeliness of the research question (H_1_) being true in their given example. Homeopathy is, at least in Sweden where the study was conducted, a controversial alternative treatment, which does not convey with conventional science (23, 24). The results imply that statistical inferences may be biased by previous contexts, in the sense that facing a controversial test result makes you more sceptical of drawing firm conclusions based on statistical test results. These findings were not a result of primary aim of the study, and thus, they should be viewed as tentative, in need to be confirmed in future research.

### Limitations

This study has some limitations. The study samples are small and were obtained by reaching out to PhD students and persons believed to be interested in statistics. The majority of persons contacted did, however, not respond, and responders are, thus, believed to be extra interested in participating in this kind of research. This means that the results are difficult to generalise. On the other hand, if the participants have a higher interest in inferential statistics, they may also have higher knowledge in the field, suggesting that inferential mistakes are more common in general than these results show.

There is no consensus in how interpretations of statistical test results should be investigated. Our outcome questions have been used previously (19), and they use a rather informal language to assess what the results ‘say’ about the truthfulness and probability of the hypotheses, H_0_ and H_1_. The informal wordings might influence the way the questions are understood, and that a statement such as ‘it shows that H_0_ is improbable’ is not seen as a probability statement. If so, respondents might have responded to the questions in a broader, non-statistical way. However, everyone participating in the survey was informed that it was about interpreting statistical test results. Furthermore, it is wrong, even in a non-statistical way, to infer that a low *P*-value/statistically significant findings implies that the nollhypotesen is improbable. This has been stressed by several authors (8, 18, 19) and was also one of points stressed in the ASA-statement. *P*-values do not measure the probability that the studied hypothesis is true. A *P*-value is a statement about the data in relation to a specified hypothetical explanation and is not a statement about the explanation itself (12).

All data were self-reported. There was no possibility to control if participants responded truthfully about their education and skills. However, we do not have any reason to believe that respondents lied or exaugurated, and the distribution of characteristics seems plausible. It is important to note that our final study populations are results of selection. They were invited to participate because of being either a doctoral student (doctoral students) or being a paid member in associations specifically devoted to statistics and research methodology (statisticians). It is further reasonable to assume that those who participated were interested in the question about the interpretation of research results. For these reasons, it is not advisable to generalise the prevalence of findings to other populations.

The main results in this article are descriptive, providing the proportion or respondents responding on various ways. We did, however, also perform hypothesis tests, which begs the question about another relevant limitation or objection to this study. This study is about correct inferences of statistical null hypothesis testing, and one of the main conclusions is that inferential mistakes persist. This conclusion is, however, also a result of statistical inferences based on hypothesis tests. In accordance with the limitations of the chosen method, we cannot claim to have proven, or shown that it is probable, yet the results add to the evidence against the null. However, the conclusion that statistical inferential mistakes persist is also well supported by the descriptive results.

## Conclusion

This study shows that inferential mistakes of *P*-values and statistically significant test results persist also among persons who have substantial statistical education and who work professionally with statistics. The results further provide preliminary evidence that statistical inferences can be biased based on the context. This finding needs to be confirmed in future studies.

## Disclosure statement

The authors report no conflict of interest.

## Funding

None.

## Notes on contributors

***Per Lytsy***, MD, PhD, works as a medical adviser at the Swedish Agency for Health Technology Assessment and Assessment of Social Services (SBU) and is associated researcher at the department of public health and caring sciences, Uppsala university and at the department of clinical neuroscience, Karolinska Institutet.

***Mikael Hartman***, is a licensed nurse and independent researcher.

***Ronnie Pingel***, PhD, works at a senior lecturer and is head of the department of statistics, Uppsala university.

## ORCID

Per Lytsy https://orcid.org/0000-0003-1949-6299

Ronnie Pingel https://orcid.org/0000-0002-4140-1981
